# Volume and flow modulation strategies to mitigate post-hepatectomy liver failure

**DOI:** 10.3389/fonc.2022.1021018

**Published:** 2022-11-17

**Authors:** Richard Bell, Saleema Begum, Raj Prasad, Kojiro Taura, Bobby V. M. Dasari

**Affiliations:** ^1^ Department of Hepatobiliary and Transplant Surgery, St. James’s University Hospital, Leeds, United Kingdom; ^2^ Department of Hepatobiliary and Pancreatic (HPB) and Transplant Surgery, University Hospital Birmingham, Birmingham, United Kingdom; ^3^ Division of Hepatobiliary and Pancreatic (HPB) Surgery and Transplantation, Department of Surgery, Kyoto University Graduate School of Medicine, Kyoto, Japan; ^4^ Institute of Immunology and Immunotherapy, University of Birmingham, Birmingham, United Kingdom

**Keywords:** post hepatectomy liver failure, future liver remnant, flow modulation, liver resection, risk mitigation, volume modulation

## Abstract

**Introduction:**

Post hepatectomy liver failure is the most common cause of death following major hepatic resections with a perioperative mortality rate between 40% to 60%. Various strategies have been devised to increase the volume and function of future liver remnant (FLR). This study aims to review the strategies used for volume and flow modulation to reduce the incidence of post hepatectomy liver failure.

**Method:**

An electronic search was performed of the MEDLINE, EMBASE and PubMed databases from 2000 to 2022 using the following search strategy “Post hepatectomy liver failure”, “flow modulation”, “small for size flow syndrome”, “portal vein embolization”, “dual vein embolization”, “ALPPS” and “staged hepatectomy” to identify all articles published relating to this topic.

**Results:**

Volume and flow modulation strategies have evolved over time to maximize the volume and function of FLR to mitigate the risk of PHLF. Portal vein with or without hepatic vein embolization/ligation, ALPPS, and staged hepatectomy have resulted in significant hypertrophy and kinetic growth of FLR. Similarly, techniques including portal flow diversion, splenic artery ligation, splenectomy and pharmacological agents like somatostatin and terlipressin are employed to reduce the risk of small for size flow syndrome SFSF syndrome by decreasing portal venous flow and increasing hepatic artery flow at the same time.

**Conclusion:**

The current review outlines the various strategies of volume and flow modulation that can be used in isolation or combination in the management of patients at risk of PHLF.

## Introduction

Post hepatectomy liver failure (PHLF) is the most common cause of death following major hepatic resections (1) but despite recent innovations to improve outcomes following hepatic resection the incidence of PHLF has still been reported as between 1% to 35%, with a perioperative mortality rate as high as 40% to 60% in the last decade (2, 3). The most commonly used definitions include ‘50-50’ criteria which was predictive of 60-day mortality in a series of 775 hepatic resections and the International Study Group of Liver Surgery (ISGLS) consensus definition for PHLF. This describes three grades of PHLF with a mortality ranging from 0% for grade A to 54% for grade C (4).

Identification of patients ‘at risk’ is essential with risk factors including advanced age, diabetes mellitus, increased BMI, preoperative chemotherapy and underlying liver disease such as fibrosis and cirrhosis (5). However, one of the most important risk factors is an inadequate future liver remnant (FLR) volume which has been defined as FLR volume/Total Liver volume (TLV) of <25% in those with a healthy background liver and in those with background liver disease, FLR/TLV of >40% is required (6, 7). Given the significant morbidity and mortality associated with PHLF numerous strategies have been used to try and mitigate the risk of it developing. The aim is to increase the volume and function of future liver remnant and ensuring that the portal venous flow and pressures are appropriate to prevent the small for size flow syndrome.

This study aims to review the current literature available relating to flow and volume modulation of the FLR to mitigate PHLF.

## Methods

An electronic search was performed of the MEDLINE, EMBASE and PubMed databases from inception of the database to January 2022. Prospective and retrospective clinical studies that investigated strategies to increase the FLR or modulate blood flow to the liver prior to liver resection were included. Conference abstracts, letters and editorials were excluded. The following search strategy comprising MeSH headings and truncated word searches to identify all articles published relating to volume or flow modulation prior to liver resection was used: future liver remnant, post hepatectomy liver failure, portal vein embolization, embolization of the portal venous branches, hepatic vein embolization, dual vein embolization, bi-embolization, liver venous deprivation, Associating liver partition and portal vein ligation for staged hepatectomy, ALPPS, portal vein ligation. The references of included studies were also reviewed to identify additional studies.

### Volume Modulation

#### Portal vein embolization/ligation

Portal vein embolization is a well-established technique for preoperative augmentation of the FLR and has been used for approximately 30 years (8). Several different approaches have been described with either an ipsilateral or contralateral approach being most common. The main advantage of the former being the avoidance of directly puncturing the FLR and the potential complications associated with this whereas the contralateral approach is technically easier allowing straight catheterization of the right portal vein (9). However, if segment 4 requires embolization this can be challenging from a contralateral approach. Embolization of segment 4 in addition to the right portal vein has shown increased hypertrophy and increased kinetic growth rate when compared to right PVE alone (10, 11). As an alternative for the access to portal vein, trans-ileocecal approach is used occasionally. It is useful for cases where direct puncture of intrahepatic portal branches is difficult, for example, huge liver tumors (12). The combination of trans-ileocolic portal embolization with associating liver partition with portal vein ligation for staged hepatectomy (ALPPS) has also been reported, which does not require dissection of the hilum in the first stage and may be good in the context of major hepatectomy for hilar cholangiocarcinoma (13).

PVE is generally considered to be a safe procedure with low rates of morbidity and mortality with the most common complication being a ‘post-embolization syndrome’ characterized by fever, abdominal pain and elevation of liver transaminases (14). More serious complications are fortunately rarer and include portal vein thrombosis, hematoma and abscess formation with an incidence of each being <1%. In a large systematic review non-target embolization occurred in 0.6%. In the same study complications led to un-resectability in only 0.4% of patients with an overall procedure mortality rate of 0.1% (15, 16).

Portal vein embolization is associated with high rates of technical success with rates >95% consistently reported. Hypertrophy of the FLR is reported as a mean of 38 – 49% in systematic review and meta-analysis with hypertrophy of over 50% in the context of additional segment 4 embolization. In the context of fibrosis or cirrhosis a rate of hypertrophy more than 10% can generally be considered safe to proceed with resection whereas in a normal liver this is more than 5% (17, 18). The majority of FLR hypertrophy occurs within the first 3-4 weeks with the maximum volume usually achieved by about 6 weeks.

Whilst FLR hypertrophy with PVE is effective perhaps the most frequent limitation to its use is that of disease progression whilst awaiting adequate hypertrophy. It has been hypothesized that tumor progression may be accelerated by the release of growth factors released as a consequence of PVE (19). However, longer term follow-up of patients undergoing liver resection with versus without PVE is conflicting with some studies demonstrating no difference in hepatic recurrence or overall survival up to 5 years (20). Whilst other show inferior survival (21, 22). Whilst there is variation in the reported rates of successful resection following PVE commonly approximately 70-75% of patients ultimately complete the treatment sequence (21). In a systematic review and meta-analysis of 44 studies including 1791 patients the overall morbidity rate was 21.7% with a mortality of 3.3%. Primary liver failure (0.4%) or liver failure in combination with multiorgan failure (1.2%) was the cause of death in over 50% of cases (15).

Portal vein ligation (PVL) is an alternative to PVE and most frequently used in the context of two stage hepatectomy (TSH) whereby the FLR is cleared of tumor along with ligation of the right portal vein to induce hypertrophy in the FLR. PVE and PVL have been compared in meta-analysis with a comparable morbidity and mortality profile along with similar percentage increase in FLR. There was also no difference between the groups with regards to disease progression precluding liver resection (23).

#### Associating liver partition with portal vein ligation for staged hepatectomy

To try and mitigate the progression of disease whilst waiting for adequate hypertrophy various strategies have been developed to promote a more rapid hypertrophy thus allowing resection to take place at an earlier stage. The initial ‘classic’ ALPPS demonstrated FLR hypertrophy rates of 75% allowing the second stage of resection to take place after a median of 9 days (24). Although this new approach showed very rapid hypertrophy this was offset by a significant morbidity and mortality with data from the International ALPPS Registry showing a 90-day mortality of 12% and major complication rate (Clavien-Dindo ≥3b) of 27% (25). Outcomes for elderly patients and those undergoing resection for hepatocellular carcinoma, peri-hilar cholangiocarcinoma and intrahepatic cholangiocarcinoma were even worse leading to the adoption of ALPPS for primarily colorectal liver metastases (26). Due to the significant morbidity associated with ALPPS various modifications were proposed to the original technique to try and improve outcomes. These include partial ALPPS (partial transection with PVL), hybrid ALPPS (complete transection with PVE between 2 stages), RALPPS (radiofrequency ablation of transection line with PVL), mini ALPPS (partial transection with PVE *via* inferior mesenteric vein) and laparoscopic ALPPS (27, 28). In addition to modifications of the technique, timing of the second stage is also important with a slight delay favored by some units. With the second stage performed after about 2 weeks (29). The recent ‘benchmarking’ of ALPPS using the registry of 1036 patients identified completion of stage 2 >96%, PHLF after stage 2 <5%, overall morbidity for stage 1 and 2 of <65% and major complications <38% and the 90-day mortality of <5% indicating similar outcomes to other types of major hepatectomy (30).

Despite the high morbidity and mortality associated with ALPPS there is evidence from the Scandinavian LIGRO Trial of increased resectability rates when compared with those undergoing PVE although the 90-day mortality remained high particularly in the context of the known high recurrence rate (31). ‘Salvage’ ALPPS has also developed in the setting of inadequate hypertrophy following PVE with mean FLR hypertrophy for this approach being between 57-65% (32–34)

#### Combined portal and hepatic vein embolization

Given the concerns regarding disease progression whilst waiting for adequate hypertrophy with PVE and the high morbidity and mortality associated with ALPPS alternative methods of modulating the FLR have been sought. Initially reported by Hwang et al. in 2009, embolization of the portal vein and then hepatic venous outflow sequentially after several weeks has been shown to be safe and effective. Initial reports show an increase in FLR from 35% pre-PVE to 40% 1-2 weeks after PVE and finally 44% 2 weeks after hepatic vein embolization (HVE) (35). Given the promising initial reports the technique has been further modified to perform embolization of the portal and hepatic veins simultaneously. This has been given multiple different names including liver venous deprivation (LVD), bi-embolization, dual vein embolization (DVE) and Radiological Simultaneous Porto-hepatic Vein Embolization (RASPE) (36–38). Although retrospective in nature the available studies demonstrate that DVE is a safe, low morbidity procedure with the most frequently cited complication being a ‘post-embolization’ syndrome characterized by fever and abdominal pain with treatment being supportive. Studies comparing the hypertrophy of the FLR between PVE and DVE show a superior percentage hypertrophy with DVE, 59% versus 48% (p=0.020) and 61% versus 29% (p=<0.001) in two of the larger studies (38, 39). Similarly the kinetic growth rate associated with DVE also appears to be superior to PVE alone with one study showing a rate of 3.5 versus 2.5 (sFLR/week) (p=<0.001) with kinetic growth rate being an important predictor of postoperative morbidity and mortality after liver resection in those with a small FLR (40). A recent meta-analysis showed that significantly more patients progress to liver resection following DVE 11% versus 24% (p=0.009). In that study only 3/20 patients didn’t progress to surgery due to inadequate FLR whereas 23/79 following PVE alone still couldn’t undergo surgery due to inadequate FLR. The most common other reason for not undergoing surgery was disease progression. Following liver resection rates of major complications appeared lower as did the incidence of PHLF with DVE, 13% versus 22% (p=0.13) although this did not reach statistical significance. Post-operative mortality was also improved following DVE (41). Whilst several randomized studies are currently in progress comparing PVE to DVE (DRAGON 1 –Training, Accreditation, Implementation and Safety Evaluation of Combined PVE/HVE – ClinicalTrials.gov NCT04272931 and HYPER-LIV01 the outcomes from retrospective studies, and meta-analysis of these, suggests that DVE is associated with improved hypertrophy and perhaps lower complications, particularly PHLF than PVE alone (42).

Only one retrospective study has directly compared outcomes following DVE to ALPPS. That study involved 209 patients of whom 124 had DVE and 85 underwent ALPPS. This showed that hypertrophy was greater with ALPPS with higher rates of surgical resection (72% versus 91%, p=<0.001). Although operative duration, blood loss and length of stay were better with LVD there was no difference in major complications or mortality (43). While the studies have demonstrated a greater increase in FLR volume with LVD, PHLF was encountered in 13% of the patients and it must be remembered that volume doesn’t necessarily equate to function. Dynamic 99mTc-mebrofenin hepatobiliary scintigraphy with single photon emission computed tomography is one method that has been used to quantitively assess liver, and FLR, function (44). The FLR will be assessed not only for change in volume but also function using 99mTc-mebrofenin SPECT-CT and will add considerably to the evidence base for PVE/DVE (43). Guiu et al. investigated the impact of PVE and LVD using 99mTc-mebrofenin SPECT-CT measuring function and volume at day 7, 14 and 21 post procedure. FLR function and volume was significantly greater at all time points with LVD as opposed to PVE (45).

#### Trans-arterial chemoembolization combined with portal vein embolization

Hypertrophy of the FLR is much more variable in patients with chronic liver disease undergoing liver resection increasing the risk of PHLF. This is also the case following PVE in this patient group. The addition of TACE prior to PVE for patients with HCC has been demonstrated to increase FLR hypertrophy compared to PVE alone (46). The additional benefit of this approach is the arterial embolization provides treatment to the tumor in the embolized lobe reducing the risk of disease progression. FLR hypertrophy using this sequential technique ranged between 7 – 56% with typically 2 – 3 weeks interval between the two procedures (47). The main concern with this approach is that of liver infarction and therefore care must be taken to embolize as distally as possible as well as modifications to the technique used. Some have proposed reversing the sequence of procedures with PVE performed prior to TACE suggesting that the degree of hypertrophy is dependent on the period of time between PVE and hepatectomy and that performing PVE first may reduce the likelihood of liver infarction and abscess formation (47).

It seems likely that there will be a place for all different methods of volume modulation for those with a predicted low FLR prior to major hepatectomy. PVE and two stage hepatectomy with PVL are well established with a good safety profile and will continue to be used routinely by a large number of centers. As experience with newer techniques such as DVE increases and the evidence base grows for this approach it is feasible that this will become the predominant method for FLR volume modulation for colorectal liver metastases as well as primary liver cancers. It might also be that the role of PVL will be limited to the setting of ALPPS. The role for ALPPS is harder to predict with opinion still divided over its role. Whilst acceptable results have now been demonstrated in high volume centers (30) there remains a reluctance by many to adopt this as the initial method for FLR modulation preferring to use it as a ‘salvage’ procedure after inadequate hypertrophy with PVE/DVE. Traditionally ALPPS has been reserved for patients with CRLM due to the initial very high morbidity and mortality associated with its use in hepatocellular carcinoma and cholangiocarcinoma. More recently the role of ALPPS for HCC and both perihilar and intrahepatic cholangiocarcinoma has been re-explored with outcomes comparable to more traditional approaches most probably related to modification of the technique with partial ALPPS and a minimally invasive approach used to reduce surgical stress (48–50).

### Flow modulation

Following major hepatic resections, the rise in portal venous flow in the presence of low remnant volume culminate into small for size flow (SFSF) syndrome (51). The key mechanism is the whole maintained portal flow diverted to remnant liver causing sinusoidal congestion and damage to endothelial lining leading to hemorrhage and architectural disruption and hepatocyte injury. High portal vein pressures (PVP) also result in hepatic artery buffer response by reducing hepatic artery pressures leading to ischemic biliary injury and cholangitis (52) These changes are irreversible and liver parenchyma loses its capability to regenerate. The incidence of SFSF is directly dependent on transhepatic portal vein and hepatic artery flow, portal pressures and volume of remnant liver. Portal venous flow of 250ml/min/100g is considered as the upper limit for SFSF syndrome by Troisi et al. (52). Thus, surgical techniques that decrease portal vein flow/100g and portal vein pressure, as well as increase hepatic artery flow/100g following extended hepatic resections can prevent the occurrence of SFSF (53). **Table 1** summarises papers describing flow modulation strategies to mitigate SFSF following liver resection. 

**Table 1:** Studies reporting portal flow modulation techniques after liver resection to prevent PHLF.

Study	Procedure and indication	Number of patients	Intervention	Condition	Outcome	Outcome parameters
Golriz et al. (52) (Animal model)	Extended liver resection	40 pigs	Portocaval shunt		Improved hepatic artery flow	PVP reduced from 16 ± 1.29 to 9.9 ± 0.66 mmHg and HAP increased from 17.77 ± 2.8 to 24.07 ± 2.08 ml/min/100 g
Arakawa et al. (55) (Animal model)	90 percent hepatectomy	25 rats	Splenectomy		Improved liver regeneration	Hemeoxygenase-1 and its messenger RNA expression increased
Ren et al. (61) (Animal model)	50%, 60%,70% and 90% hepatectomy	160 rats	Splenectomy		Improved liver regeneration and liver functions	Increased DNA synthesis and proliferation cell nuclear antigen.
Hammond et al. (70) (Animal model)	80 percent hepatectomy	24 pigs	Portocaval shunt and terlipressin		Increased hepatic artery flow and reduced the incidence of PHLF.	HAF increased to 73ml/min from 40ml/min.
Jo et al. (71) (Animal model)	90% hepatectomy		Terlipressin		Optimize liver regeneration and improved survival	PVP reduced to 5.8 ± 1.1 from 7.7 ± 2.2 mmHg one hour after hepatectomy
Rhaiem et al. (72)	Major hepatectomy	10 patients	Somatostatin	Cirrhosis	Effective portal flow modulation	3 mm median reduction in PVP
Takamatsu et al. (73)	Right hepatectomy for HCC	1 patient	Splenectomy	Cirrhosis	Reduced the incidence of PHLF	PVP reduced from 32 to 23
Kohler et al. (74)	Major liver resection for HCC, IHCC and CRLM	75 patients	Terlipressin	Cirrhosis, Child Pugh A and B	No difference	
Abbas et al. (75)	Major liver resections Indication not mentioned	42 patients	Terlipressin	Cirrhosis, Child A and B	No difference	
Mahdy et al. (76)	Major liver resections	25 patients	Terlipressin	Not mentioned	Improved intraoperative hemodynamic and blood loss	PVP reduction from 17.88 ± 7.32 to 15.96 ± 6.55
Li et al. (77)	Major liver resections	65 patients	Terlipressin	Cirrhosis	Reduced the incidence of PHLF and ascites.	Ascites volume decreased from 730ml to 350ml and PVP reduced from 15.8 ± 2.6 to 14.3 ± 2.9
Pei et al. (78)	Major and minor liver resections	184 patients	Splenectomy	Cirrhosis, Child A and B	Significantly improved liver functions	Improved liver functions in Child B patients
Carrapita et al. (79) (Animal model)	85% hepatectomy	48 rats	Splenic artery ligation		Increased hepatocellular viability and regeneration	Increased viability of cells and decreased oxidative stress.

Portal flow modulation was initially applied in living donor liver transplantation. Techniques including portal flow diversion, splenic artery ligation and splenectomy are employed to reduce the risk of SFSF syndrome by decreasing portal venous flow and increasing hepatic artery flow at the same time (54).

### Splenectomy

Splenic blood flow contributes to 25-30% of the total portal flow which may rise up to 50% in portal hypertension and plays a crucial role in portal overpressures following living donor liver transplantation and major hepatic resections. The role of splenectomy in portal flow modulation was first studied in rodent models (55). Splenectomy increases vascular compliance of graft, and hepatic serotonin levels which improve hepatic perfusion through its vasodilatory effect. Serotonin provides protection to the graft by increasing microcirculation and accelerates liver regeneration by stimulating endothelial cells to release vascular endothelial growth factor (56, 57). Simultaneous splenectomy reduced hypersplenism and prevented graft congestion from excessive portal flow in the first outcome report of six cases after left lobe living donor liver transplantation, and excluding splenectomy was an independent risk factor for SFSF in these patients (58). Patient survival rates were significantly higher in patients with a PVP ≤ 15 mm Hg than those with PVP ≥ 15mm Hg in a study by Kaoido et al. (59). Similarly, Kyoto group found that failure of flow modulation (which they achieve with splenectomy) to maintain a PVP ≤ 15mm Hg is associated with SFSF syndrome and early graft loss (60).

The role of splenectomy in flow modulation and preventing SFSF syndrome is well described in LDLTs; however, its role in extended hepatectomies is only described in animal studies. Splenectomy significantly improved liver functions, and enhanced DNA synthesis and proliferation of cell nuclear antigens to facilitate liver regeneration in rats undergoing major hepatectomies (61). Similarly, Arakawa et al. reported reduced hepatocyte damage and improved survival after 90% hepatectomy with splenectomy in rats (55).

### Splenic artery ligation

Splenic artery ligation was used in LDLT to prevent the risk of thrombocytopenia associated with splenectomy, however, later it was reported as an effective way to reduce the PVP and increase hepatic artery flow by Troisi et al. (62). Therefore, it is used as an alternate to splenectomy in reducing PVP and PVF. Shimada et al. showed that patients who underwent splenectomy after LDLT had better graft function and survival at one year (91.2% vs 77.9%) compared to splenic artery ligation, indicating the inferiority of later in flow modulation (63). Moon et al. described splenic devascularization as an alternative to splenectomy in selected LDLT recipients where SA and right gastro epiploic arteries and short gastric arteries were ligated and divided. In this small retrospective study, authors reported a better safety profile of this method (64).

Non-surgical ways for splenic artery ligation like splenic artery embolization have been described in small case series and case reports for flow modulation in LDLT showing promising results with less procedure related morbidity than (65). In context of PHLF, convincing data is still lacking to consider splenic artery ligation as a therapeutic option for flow modulation.

### Portocaval shunts

The role of portocaval shunts in preventing SFSF from portal hyper-perfusion has been described for LDLTs. The decompression of portal system can prevent sinusoidal congestion and graft dysfunction in experimental models (66, 67). In clinical settings, hemi-portocaval shunts have shown better patient and graft survival (68). In a series of 13 patients undergoing adult to adult LDLT, Troisis et al. found a significant reduction of portal vein flow among the hemi-portocaval shunt group compared to the group without graft inflow modulation (190 ± 70 ml/min/100 g liver v/s 401 ± 225 ml/min/100 g liver; p < 0.001). It is important to note that excessive diversion of portal flow into systemic circulation can lead to steal syndrome which can cause graft ischemia. Therefore, Troisi et al. recommend measuring portal pressure and calibrating the size of shunt to avoid steal phenomenon (69). Given the similarity in underlying flow dynamics of LDLT grafts and remnant livers post hepatectomy, shunts can be an attractive future direction in preventing PHLF.

### Pharmacological interventions

Although surgical techniques like splenectomy, splenic artery ligation and creation of shunts in animal models have shown promising results in portal flow modulation and decreasing the risks of PHLF, the procedure adds to the complexity of liver resection with associated morbidity. This led to the idea of exploring non-invasive options to reduce the portal venous flow and pressure using splanchnic vasoactive agents like octreotide, terlipressin and vasopressin. Historically, Tri-glycyl-lysine (terlipressin) has been used in cirrhotic patients to treat the complications of portal hypertension. Recent studies on pigs and rodents have shown marked reduction in portal venous flow and pressure and attenuation of liver injury after 80% and 90% hepatectomies (70). A study by Jo et al. demonstrated improved liver regeneration and survival with terlipressin in pigs following 90% hepatectomy with rapid and effective flow modulation (71). Similarly, due to its antioxidant and vasoconstrictor properties, somatostatin has been suggested as an experimental agent in reducing the risk of PHLF (70). Although the techniques for flow modulation have shown promising results in animal models and LDLTs, there role in reducing the risk of PHLF in clinical settings is still debatable and further studies are required to address this important issue.

## Conclusion

In conclusion, the current review outlines the various strategies of volume and flow modulation that can be used in isolation or combination in the management of patients at risk of PHLF. PVE and PVL are well established methods of reducing PHLF in those with a small FLR. ALPPS and DVE show great promise at producing a larger, more rapid hypertrophy that may allow more patients to undergo potentially curative liver resection. Methods to modulate flow to the FLR are more established in liver transplantation, and in particular live donor liver transplant, but remain largely untested in the context of liver resection and mitigating PHLF.

## Author contributions

RB and SB contributed equally and should be considered as first authors. BD: substantial contributions to the conception or design of the work; revising the draft, approval for publication. RB: substantial contributions to the acquisition, analysis, or interpretation of data for the work drafting the work and provide approval for publication of the content. SB: substantial contributions to the acquisition, analysis, or interpretation of data for the work drafting the work and provide approval for publication of the content. RP: substantial contributions to the conception or design of the work; revising the draft, approval for publication. KT: substantial contributions to the conception or design of the work; revising the draft, approval for publication. All authors contributed to the article and approved the submitted version.

## Funding

University of Birmingham contributed towards the Open Access Publication fees.

## Conflict of interest

The authors declare that the research was conducted in the absence of any commercial or financial relationships that could be construed as a potential conflict of interest.

## Publisher’s note

All claims expressed in this article are solely those of the authors and do not necessarily represent those of their affiliated organizations, or those of the publisher, the editors and the reviewers. Any product that may be evaluated in this article, or claim that may be made by its manufacturer, is not guaranteed or endorsed by the publisher.
